# Live-Cell Systems in Real-Time Biomonitoring of Water Pollution: Practical Considerations and Future Perspectives

**DOI:** 10.3390/s21217028

**Published:** 2021-10-23

**Authors:** Donald Wlodkowic, Tomasz M. Karpiński

**Affiliations:** 1 The Neurotox Laboratory, School of Science, RMIT University, Plenty Road, P.O. Box 71, Bundoora, VIC 3083, Australia; 2 Chair and Department of Medical Microbiology, Poznań University of Medical Sciences, Wieniawskiego 3, 61-712 Poznań, Poland; tkarpin@ump.edu.pl

**Keywords:** water, pollution, early warning, sensor, bacteria, algae, cell

## Abstract

Continuous monitoring and early warning of potential water contamination with toxic chemicals is of paramount importance for human health and sustainable food production. During the last few decades there have been noteworthy advances in technologies for the automated sensing of physicochemical parameters of water. These do not translate well into online monitoring of chemical pollutants since most of them are either incapable of real-time detection or unable to detect impacts on biological organisms. As a result, biological early warning systems have been proposed to supplement conventional water quality test strategies. Such systems can continuously evaluate physiological parameters of suitable aquatic species and alert the user to the presence of toxicants. In this regard, single cellular organisms, such as bacteria, cyanobacteria, micro-algae and vertebrate cell lines, offer promising avenues for development of water biosensors. Historically, only a handful of systems utilising single-cell organisms have been deployed as established online water biomonitoring tools. Recent advances in recombinant microorganisms, cell immobilisation techniques, live-cell microarrays and microfluidic Lab-on-a-Chip technologies open new avenues to develop miniaturised systems capable of detecting a broad range of water contaminants. In experimental settings, they have been shown as sensitive and rapid biosensors with capabilities to detect traces of contaminants. In this work, we critically review the recent advances and practical prospects of biological early warning systems based on live-cell biosensors. We demonstrate historical deployment successes, technological innovations, as well as current challenges for the broader deployment of live-cell biosensors in the monitoring of water quality.

## 1. Introduction

Water resources free of chemical pollutants are paramount for the protection of the biosphere, as well as a sustainable supply of water that is safe for human use and the production of food. The increasing anthropogenic pollution of the aquasphere and the impact of accelerating climate changes are driving shortages of potable water of a suitable quality for large urban populations and remote rural communities alike [1]. The contamination of water with industrial chemicals predominantly arises from long term, cumulative processes. However, it can also occur rapidly following industrial accidents, deliberate and illegal waste disposal and emergent threats associated with chemical terrorism [1,2,3,4,5,6]. Rapid pollution incidents such as these cannot be predicted but have a significant potential of becoming catastrophic events, especially considering the rapidly increasing potable water shortages worldwide [1,5,6].

In this regard, an important component of source water protection programs are future online systems capable of continuously monitoring water quality and providing early warnings of developing chemical hazards [1,5,6,7,8]. They could increase the resilience of water supplies and significantly reduce the risks associated with chemical discharge emergencies. Such systems have been indicated by many governmental regulations including the US Safe Water Drinking Act (SWDA) that highlights the importance of source water assessment and protection programs safeguarding water supplies [5,7]. The early warning systems can be used to provide timely information on the quality of the source water so that knowledgeable decisions can be made concerning treatment and operation decisions [6].

There have been several developments in innovative technologies aimed at the water industries’ needs for the continuous monitoring of the chemical and physical parameters of water, including automated water sampling and remote sensors distributed in water delivery networks [6,8]. The latter can provide a multiparametric and highly automated analysis of standard water indices such as pH, turbidity, chlorine, dissolved oxygen (DO)and total organic carbon (TOC). Such technologies are becoming commonplace at many water installations across Europe, the United States and Asia [8,9]. However, despite their significant advantages in the day-to-day online analysis of standard water quality parameters, they have some major drawbacks. Namely, they are incapable of deciphering if an accidental discharge of toxic chemicals has occurred. Moreover, they cannot detect any additive and synergetic effects of toxicants on living organisms [1,2,7,8,10,11].

Recent developments of automated gas and liquid chromatography systems have attempted to at least partially address the above issues. Although such systems provide a noteworthy high degree of automation in the quantitative analysis of water chemistry, they are limited by realistic sampling frequencies, non-trivial costs of infrastructure and complex maintenance [1,2,8]. This often results in very high costs per sample. As such, the routine adoption of these technologies for the online monitoring of water quality is still largely impractical [2,8].

Developments of biosensors were also made to address water quality monitoring, in particular, for catchments of drinking water [8,12,13,14]. A biosensor is best characterised as an integrated device where a bioassay using a cell-free or a whole cell-based system provides the quantitative or qualitative detection of an analyte. The signal generated by the biological sensing part is detected and amplified by electronic circuitry, thus providing a user-friendly data readout [12,13,14,15]. The ultimate goal for biosensors is high sensitivity and specificity, defined as an ability to detect specific analytes [14,15,16]. In this regard, significant developments in cell-free biosensors that utilise immune, enzymatic as well as nucleic acid detection assays have also been demonstrated for the detection of toxicants in water [17,18,19]. Such biosensing technologies are rapid and simple to use but are often single, analyte-specific, require replacement upon detection and can be susceptible to the biofouling and abiotic parameters of water.

**Figure 1:**
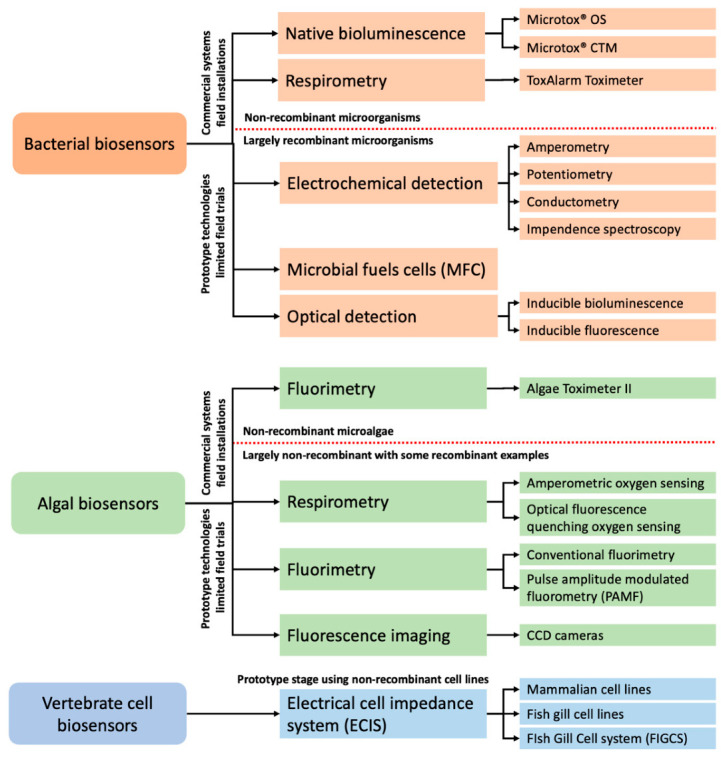
Overview of live-cell biosensing and biomonitoring systems for water quality assessment.

To address the above limitations, biological early warning systems (BEWSs) have historically been proposed [1,2,3,4,20]. Such biomonitoring technologies can address this analytical gap and supplement conventional water testing by providing continuous evaluations of water quality. This is usually achieved by the real-time evaluation of physiological parameters of suitable aquatic bioindicator species [1,2,20]. In contrast to biosensors, the BEWS technologies for online water biomonitoring applications are almost never designed with analyte-specific detection in mind. They are inherently non-specific and lack the ability to provide quantitative and qualitative chemical detection capabilities [1,2,20]. They can, however, provide continuous sensing and an early warning about a sudden alteration in the water quality parameters by monitoring alterations of the carefully established baselines of physiological parameters. Such systems predominantly rely on the real-time behavioural analysis of metazoan animals and have recently been thoroughly reviewed by Bownik and Wlodkowic [2]. BEWSs and online biosensors can, however, also be developed using single cellular organisms. Bacteria, cyanobacteria, algae and micro-algae offer, in this regard, many promising avenues for the development of real-time and near real-time water biosensors (Figure 1) [12,13,14,15,16,21]. Biosensors utilising intact and functional cells are commonly referred to as live-cell biosensors. The measurement of diverse cell attributes such as viability, proliferation, metabolic state and many others serves as a readout to detect toxic changes in the water samples. Historically, only a handful of systems utilising live cells have been deployed as established online water biomonitoring tools. Recent advances in recombinant microorganisms, cell immobilisation techniques, live-cell microarrays and microfluidic Lab-on-a-Chip technologies open new avenues to develop the miniaturised biosensing systems capable of detecting a broad range of water contaminants [12,13,14,21].

In this work, we critically review the advances and prospects of practical applications of biological early warning systems based on live-cell biosensors that utilise bacteria, algae as well as vertebrate cell lines. We demonstrate historical successes with existing practical deployment examples and technological innovations as well as discuss current challenges for the broader deployment of live-cell biosensors in the monitoring of water quality.

## 2. Established Online Biomonitors Using Bacteria and Algae

Historically there have been only a handful of practical and successful deployment examples of online water biomonitoring systems that utilise live cells (Figure 1).

### 2.1. Online Microtox System

Microtox-OS was initially introduced in 1990 by Siemens Environmental in collaboration with Yorkshire Water. It was based on an established AZUR bioluminescent technology, (Azur Environmental, Carlsbad, CA, USA) where the metabolic effect of toxicity is measured as a decrease in the light output from the natively bioluminescent strain of bacteria *Aliivibrio fischeri* (formerly known as *Vibrio fischeri*) [22,23,24]. Microtox-OS was an automated version of the standard, laboratory Microtox test [11,25]. The system was equipped with a rotating carousel that held 36 vials of freeze-dried Microtox reagent. Each diluted and reconstituted vial could supply up to 30 tests. The system could operate completely unattended for up to 14 days and perform repeated analysis in 20 min intervals. A light detector measured bioluminescence in the tested and reference (control) samples. The data were logged in a time-stamped file. This innovative BEWS technology was developed for applications in testing the toxicity of drinking water intake, as well as influent and effluent monitoring in wastewater treatment plants [25]. Unfortunately, despite the inherent sensitivity and established worldwide standard applications of bioluminescent *Aliivibrio fischeri* in ecotoxicity testing, the Microtox-OS was quickly discontinued. This was predominantly related to significant limitations of early automation and electronic technologies that were proven to be unreliable and expensive in day-to-day operations.

**Figure 2:**
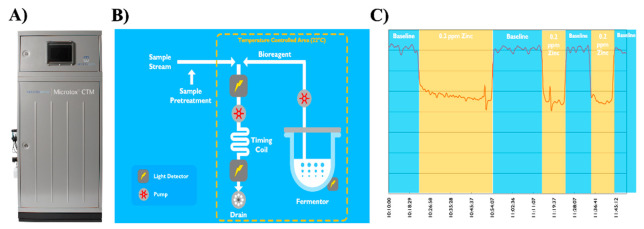
The Microtox^®®^ CTM (Continuous Toxicity Monitor, Modern Water Plc, London, UK) a commercial real-time water biomonitoring system based on a bioluminescent strain of bacteria *Aliivibrio fischeri*. (**A**) Overview of the device optimised to perform online monitoring in a continuously delivered stream of water sampled from reservoirs, water supply systems and water treatment plants; (**B**) Technical diagram depicting major components of the Microtox^®®^ CTM. Note an automated on-board fermenter that provides continuous culture of bioluminescent strain of bacteria *Aliivibrio fischeri*. The system can automatically perform one measurement every ten seconds for up to four weeks without any operator involvement, (**C**) Example response data with a reference toxicant (Zinc, 0.2 ppm). Source data provided by and used with permission from Modern Water Plc (London, UK, www.modernwater.com, accessed on 1 October 2021).

Interestingly, recently, this early concept experienced a revival and modernisation with the latest microelectronic technologies. Namely, the Microtox^®®^ CTM (Continuous Toxicity Monitor) was re-developed by Modern Water Plc (London, UK) to enable real-time biosensing (Figure 1 and Figure 2). The modern system is equipped with an automated on-board fermenter that provides a continuous culture of a bioluminescent strain of bacteria *Aliivibrio fischeri* instead of the previous carousel of freeze-dried vials with Microtox reagents (Figure 2). This enables Microtox^®®^ CTM to automatically perform one measurement every ten seconds for up to four weeks without any involvement from the operator. Furthermore, modern electronics and automation systems allow for significant advancements in reliability and rugged operational deployment. The sensitivity of Microtox^®®^ technology has been validated with a range of toxicants such as heavy metals, insecticides, fungicides, herbicides and diverse industrial chemicals (Figure 2). Reportedly, the system can also detect synergistic toxicity effects in complex mixtures of chemicals.

### 2.2. ToxAlarm Toximeter

This system was developed by LAR Process Analysers AG (Berlin, Germany) and is an example of automated BEWS based on bacterial respirometry in accordance with the ISO 9509:2006 standard (Figure 1) [11,25]. The principle of ToxAlarm Toximeter is the measurement of oxygen consumption by nitrifying bacteria [25]. The bacterial solution is automatically dosed from the fermenter and injected into a measurement cell where it mixes with an injected water sample. The measurement cell is equipped with a very sensitive oxygen sensor. Nitrifying bacteria consume oxygen from the conversion of ammonia into nitrate, and in the presence of toxic chemicals, this metabolic process is inhibited, thus reducing the overall consumption of oxygen.

The system is equipped with an automated on-board fermenter that provides a continuous culture of nitrifying bacteria. The fermenter can reportedly operate unattended for up to two weeks and after that period, the system only requires the replenishing of bacterial media. Each toxicity testing measurement lasts approximately 5 min; hence, the system can be classified as a near real-time BEWS technology. The ToxAlarm Toximeter is reportedly suitable for the online monitoring of surface and ground water, as well as catchments of drinking water.

One of the most notable examples of its deployment has been extensive evaluation on the Rhine and lower Main rivers as part of large-scale and continuous water biomonitoring programs in drinking water catchments [11,25]. This program was established by the German Commission for the Protection of the Rhine in 1990. Interestingly, a report by the Working Group of the German Federal States on Water Problems (LAWA) that provided extensive sets of evaluations and recommendations on the deployment of continuous biomonitors for the monitoring of surface waters highlighted some caution with regard to the ToxAlarm system [11,25]. It was observed that during continuous operation, diverse native bacterial populations were routinely introduced from the test water if no pre-filtration was conducted. Their growth in the tubing necessitated an enhanced daily maintenance regimen with manual rinsing and sterilisation of the system, making it cumbersome for the online monitoring of surface waters [25]. The new version of the technology is equipped with a pre-filtration stage, thus, alleviating many problems associated with the previous generations of the system.

### 2.3. Algae Toximeter II

**Figure 3:**
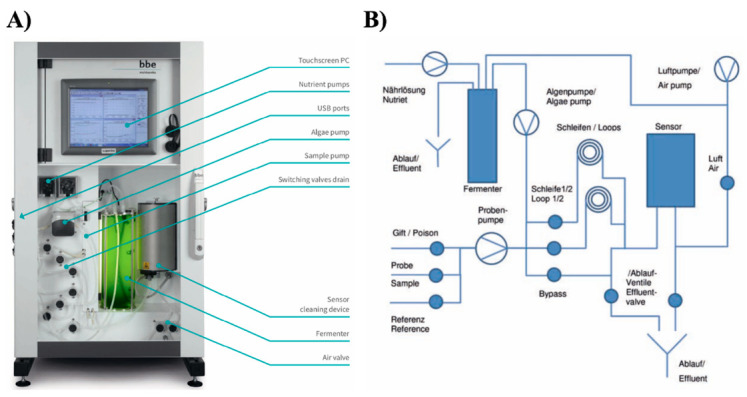
The Algae Toximeter II (bbe Moldaenke GmbH, Germany) is a commercial water biomonitoring system utilising Chlorella vulgaris microalgae as a bioindicator organism. (**A**) Overview of the device, depicting major functional components. Note an automated on-board photobioreactor/fermenter that provides continuous culture of microalgae. Analysis of each water sample takes approximately 45 min and, thus, the Algae Toximeter II is an example of time-resolved but not real-time water sensing technology; (**B**) Technical diagram of the system with its main functional components. Source data provided by and used with permission from bbe Moldaenke GmbH (http://www.bbe-moldaenke.de (accessed on 1 October 2021)).

The advances in microelectronics and fluorimetry have fuelled the development of commercial BEWSs such as an Algae Toximeter II (bbe Moldaenke, http://www.bbe-moldaenke.de (accessed on 1 October 2021)) (Figure 1 and Figure 3) [26]. It is based on a successful predecessor (Algae Toximeter I) that was time tested during continuous operation in many installations across Europe since 1995. The updated technology has been specifically designed to perform monitoring of water from drinking water purification and distribution, as well as effluents from wastewater treatment plants [11,25,26]. It can also be deployed to perform environmental monitoring in rivers and lakes providing the evaluation of remediation processes.

The Algae Toximeter II is equipped with a computer-controlled and operated photobioreactor that automatically cultures microalgae *Chlorella vulgaris* as bioindicator organisms (Figure 1 and Figure 3). They are widely used in water toxicity testing according to the ISO 8692:2012 standard [26]. The raw water sample pumped into the device is first evaluated from the perspective of concentration and activity of naturally occurring algae. For this purpose, fluorometric reference analysis estimates the total chlorophyll content in the sample as well as the concentration of different algae classes such as green, blue-green, brown (diatoms and dinoflagellates) algae and cryptophytes [26]. Subsequently, a precisely measured amount of cultured *Chlorella* cells is added from the photobioreactor to the measuring chamber. A physiological state of the microalgae upon exposure to a water sample is estimated using a direct chlorophyll fluorescence measurement (Genty method) [26,27]. Its principle is based on the fact that the Photosystem II emits light in the red spectrum range (685–700 nm) upon illumination with a 488–532-nanometer wavelength stimulus. The intensity of red emission is, thus, recorded and reflects the photosynthetic activity. The exposure to chemicals inhibits the photosynthetic activity compared to the controls and serves as an indicator of toxicant presence. The latter activates an alarm above a pre-defined threshold [26]. Each testing phase takes approximately 45 min, hence the Algae Toximeter II is an example of a time-resolved but not a real-time water sensing technology.

According to the manufacturer, microalgae demonstrate higher sensitivity to herbicides, aromatics, halogenated aromatics, chlorinated hydrocarbons, substituted organic acids, some heavy metals and surfactants than *Daphnia magna* and fish that are used in many behaviour-based real-time biomonitoring systems [26]. Thanks to its sensitivity and extensive validation, this technology has been widely deployed in many installations across Europe, the United States, Canada, Korea and China, with applications ranging from dam monitoring, wastewater treatment testing and waterway health assessment to online monitoring of both intake and treated drinking water supplies [26,28].

## 3. Automated Flow Cytometry and Online Fluorimetry

Apart from abiotic toxicants, the contamination of water with bacterial populations is highly variable in many regions. The most significant daily health threat worldwide is the possible pollution of drinking water catchments with enteric pathogens [29,30]. In this regard, there is a notable lack of suitable real-time detection technologies that could be used in early warning strategies [31]. The introduction of bacteriological online sensing systems, although not directly related to the detection of toxic chemicals, can prospectively allow us to supplement abiotic BEWSs and offer considerable potential for multi-parametric early warning water biomonitoring systems.

Flow cytometry (FC) has become the gold standard in the multi-parametric analysis of diverse human and animal cells, bacteria and even the development of rapid algal cytotoxicity assays using fluorescent viability probes [32,33]. Recently, FC has gained significant acceptance in the analysis of bacteriological water quality because it can provide a simultaneous determination of the total cell count (TCC) and bacterial viability using cell permeable nucleic acid stains such as SYTO and SYTOX [34]. Moreover, online flow cytometry has recently found its noteworthy application in an assessment of microbial dynamics in groundwater used for drinking water catchments [35]. This innovative system enables an automated 15-min interval water sampling and flow cytometric analysis of bacterial populations in water for up to 14 consecutive days.

Apart from the above example, FC has also been used to assess the ecotoxic impact of diverse pollutants on microbial and picoplankton communities in field deployments. Fully automated flow cytometry systems such as CytoSense, CytoBuoy and CytoSub (CytoBuoy b.v., Woerden, The Netherlands) provide, in this context, innovative capabilities for in situ, as well as near real-time monitoring of chemical impacts on pico-, nano- and microphytoplankton. The CytoSense system has been continually deployed since 2013 on the river Meuse as a part of the early warning system for assessing the quality of drinking water catchments.

The online fluorimetry, similarly to flow cytometry, has also been postulated as a promising avenue to provide a continuous evaluation of the bacteriological water quality [36,37]. In particular, the tryptophan-like fluorescence (TLF) (peak excitation/emission, 280/350 nm, respectively) has been reported to correlate well with water microbiological endpoints such as biological oxygen demand (BOD), chemical oxygen demand (COD) and total organic carbon (TOC) [38,39]. Importantly, recent studies have indicated that drinking water contaminated with faecal coliforms can have significantly higher TLF than uncontaminated water [38]. Overall, online fluorimetry appears as a promising future technology for a highly automated assessment of bacteriological risk in drinking water sources [37].

There are major technology challenges for automated online flow cytometry and fluorimetry. There is a considerable need for further engineering to develop systems that are robust with reliable automation but at the same time feature user-friendly operation, a low cost to analysis ratio, as well as being equipped with automated data processing and mining. There are also other significant variables that can negatively interfere with online fluorimetry analysis, such as the impact of temperature, the turbidity of raw water, the biofouling of detection elements, as well as the significant build-up of inorganic deposits such as iron ions that often hamper the measurements in long-term installations [36].

## 4. Bacterial Biosensors

Bacteria are prokaryotic microorganisms characterised by a lack of a membrane bound nucleus and intracellular organelles. Most bacteria in waterways and sediments can be classified as destruents, since they are responsible for the recycling of organic matter in the ecosystem [25]. Bacterial-based biosensors are by far the most popular technologies prototyped for toxicity and water quality testing (Figure 1) [12,13,14,15,40]. Furthermore, out of the three existing commercial whole-cell online biomonitoring systems, two are based on the implementation of bacteria-based detection. A plethora of species of bacteria were explored in water biosensing and biomonitoring prototypes, but the most popular are *Escherichia coli*, *Aliivibrio fischeri*, *Photobacterium phosphoreum* and multiple species from the genus *Pseudomonas* sp. and *Flavobacterium* sp. [12,13,14,16].

Live bacteria systems can employ diverse endpoints for the real-time detection of chemicals including a loss of cell viability, an inhibitory impact on metabolic activities, changes in electrochemical potential, inhibition of selected enzymatic activities, or more recently, an analyte-specific induction of florescence or bioluminescence in engineered transgenic strains [12,13,14,16,41]. Most of these technologies have been thoroughly reviewed in specific technical literature and a detailed description of all discrete features is beyond the scope of this review [12]. Below, however, we briefly outline the main classes of bacterial sensing technologies that have been proposed in the literature and discuss their practical applicability for online water biomonitoring applications.

### 4.1. Electrochemical Sensing

This type of technology has traditionally been very popular due to the low cost, simple design and straightforward implementation in portable applications [12,42]. Electrochemical sensing is also advantageous for online sensing since it is a label-free technique that does not require any staining with fluorescent probes or optical elements, that are usually susceptible to damage or significant fouling. The measurable output of electrochemical sensing can be the current (amperometry), potential (potentiometry) or electrical conductivity of the solution (conductometry, impedance spectroscopy) [12,42]. These technologies can utilise both native or genetically engineered strains of bacteria that can be immobilised on the specially modified surfaces or suspended in solution.

Amperometric principles measure an electric current output that occurs when electrons are exchanged between the sensing electrode and the microbial population that metabolic activities change the oxidation state of the electrode [43,44,45]. The measured signal is proportional to the amount of the redox changes occurring at the electrode and, hence, is proportional to the metabolic activities of the microorganisms [43]. The most employed variation of this technology is chrono-amperometry, where changes of the current output are measured as a function of time. Examples of amperometry sensors include prototypes for the detection of neurotoxic pesticides, heavy metals as well as phenolic and non-ionic surfactants [12,44,46,47].

Potentiometric biosensors operate on the principle of measuring the electrical potential difference between working and reference electrodes [45,48,49]. The microbial technologies of this class usually consist of modified electrodes that measure pH changes or the production of carbon dioxide by microbes [50]. Examples include bacterial potentiometric biosensors developed for the detection of the cephalosporin group of antibiotics, monitoring trichloroethylene in wastewater and organophosphate insecticides [12,50,51,52,53].

Conductometry technologies measure changes in the electrical conductivity (or resistivity) of the solution [54]. Those measurements are proportional to the analyte concentration when an electric potential (constant or sweeping) is applied between two opposing electrodes. Bacterial biosensors using this method usually detect global changes of metabolites produced by the bacterial culture because they modify the overall conductivity of the media [54,55]. Examples include prototypes demonstrated for the detection of sulfuric acid, chlorinates and heavy metals in waters [56].

Electrochemical impedance spectroscopy (EIS) measures impedance changes between a pair of electrodes immersed in a medium. The impedance is defined as the total resistance of a conductive medium [57]. This technology is also commonly referred to as impedance microbiology [57,58,59]. It is used to enumerate the density of microorganisms using an increase in the electrical conductance and capacitance of the culture media occurring as the bacteria proliferate. The increase in conductance and capacitance translates into a decrease in the measured impedance values [57,58]. Importantly, this technique not only detects the number of micro-organisms but also measures their relative metabolic activity [57,58]. The principles of this technology date back to 1899 and the seminal discovery by Stewart that the growth of bacteria changes the conductivity of the media [60]. The progress in the microelectronics and computer-controlled laboratory instrumentation from the early 1980s led to the development of many commercial applications of impedance microbiology, especially in the food and dairy industries [61,62]. The impedance bacterial biosensing has also been demonstrated in estimating pathogenic bacterial loads in water quality testing [63,64]. Commercial high-throughput impedance microbiology systems include Rapid Automated Bacterial Impedance Technique (RABIT; DonWhitley Scientific, North Gosford NSW, Australia), Bactrac (Sy-ab Microbiology, Neupurkersdorf, Austria) and, a now discontinued, Bactometer (bioMérieux SA, Marcy-l’Étoile, France).

Recently, two new impedance techniques have been proposed, including one utilising immobilised bacterial cells at the electrodes, as well as impedance microflow cytometry [57]. The former technique, instead of measuring the conductivity of the medium, estimates the interfacial impedance of interdigitated arrays of electrodes. It reflects the changes at the surface of the electrodes and effects the local capacitance of the electrode/electrolyte interface due to the bacterial presence [57]. The miniaturised Lab-on-a-Chip impedance flow cytometry has only recently been demonstrated as a promising technology for the real-time online sensing of bacteria in water. This is one example of impedance-based bacterial biosensing not performing in static environments [65].

### 4.2. Microbial Fuels Cell (MFC)-Based Biosensors

This type of technology relies on exoelectrogenic microorganisms that use organic substrates as their primary metabolic energy source [41,66,67]. In MFCs, the electrochemically active microorganisms (EAMs) donate the electrons from the electron transport chain to the anode electrode’s surface [41]. Alternatively, autotrophic microorganisms as electron donors have also been demonstrated in photosynthetic microbial fuel cells (PMFC) [41,67]. The flow of electrons and, thus, the measured amount of electricity generated in the MFCs is directly proportional to the metabolic state of the EAMs [41]. In all applications, the critical prerequisite for the occurrence of the direct electron transfer (DET) is physical contact between the bacterial cell membranes and the anode. This usually requires physical or chemical entrapment or the immobilisation of bacteria cells in biofilms directly on the surface of the anodes’ substratum [41,67]. The latter has been prototyped using diverse materials such as platinum, graphite or carbon. There have been numerous of prototypes demonstrated in recent years, including single and dual chamber MFC systems [41,66,67,68].

MFC-based biosensors have been tested under laboratory conditions for applications in water quality testing including monitoring dissolved oxygen (DO), biological oxygen demand (BOD) and chemical oxygen demand (COD), and as toxicity biosensors for heavy metals, pesticides, formaldehyde and polychlorinated biphenyls (PCBs) [41,67,69,70]. Reportedly, MFCs can provide reduced operating costs compared to other types of live-cell biosensing technologies and are reasonably low maintenance while providing long-term stability [67,69].

### 4.3. Optical Sensing

Optical sensing offers significant advantages for the implementation of genetic engineering and, thus, the development of analyte and/or effect-specific strains [12,13,14]. Virtually all bacterial optical biosensing prototypes can be classed as label-free techniques since the emitted light output is based on native luminescence or recombinant DNA technology and not on exogenous chemical fluorescent stains. The outputs are bioluminescence and fluorescence employing bacterial luciferase and green fluorescent protein (gfp), respectively (Figure 1) [13,14,16].

#### 4.3.1. Bioluminescence Methods

The utilisation of bioluminescent strains has become very popular and widely used in diverse prototypes of bacterial toxicity and water quality biosensors. This method is based on the expression of the luciferase enzyme and can offer faster overall responses and higher sensitivity compared to GFP technology [16].

There are two main variations of luminescent biosensors that utilise (i) natively bioluminescent strains and (ii) recombinant DNA technology to provide inducible expressions of luciferase [14,16].

*Aliivibrio fischeri* is an example of a naturally bioluminescent bacterium that natively exhibits a constitutive and high-level expression of bacterial luciferase (lux) [14]. This species is also very sensitive to many chemical pollutants and, thus, is ubiquitously used in the patented Microtox^®®^ technology in ecotoxicology and water quality testing [16,22]. Upon exposure to toxic chemicals, a decrease in the bioluminescence emissions is measured as an output of the *A. fischeri* bioassay. This readout is not compound-specific and represents the general cytotoxic effects on bacteria [16,22]. The Microtox^®®^ CTM is a commercial online water biomonitoring system exploiting this method of sensing.

In contrast, the recombinant DNA technology provides unique abilities to create analyte and/or biological effect-specific strains of popular *E. coli* bacteria when specific promoters are fused into a plasmid with the luciferase genes [14,16]. In this construct, the promoter controls the inducible expression of luciferase [14,71]. A low baseline luminescence output increases rapidly upon sensing the target effect or analyte (*switch on*). Moreover, the inducible expression can be designed as a *switch on–switch off* system, where bioluminescence will return to basal levels upon the withdrawal of the target analyte, thus affording self-regenerating and real-time sensing abilities. The recombinant systems can utilise both bacterial (lux) or firefly (luc) luciferase genes [14,16,71]. Despite having a higher quantum yield, the firefly luciferase does, however, require the addition of an exogenous substrate called luciferin to maintain the luminescence output. The commonly used modified bacterial luxCDABE operon contains both the luxAB genes responsible for luminescence reaction, as well as the luxCDE genes encoding pathways for the regeneration of the luciferin, thus eliminating any need to provide the substrate externally [71,72,73].

The inducible, analyte-specific bioluminescent strains that respond to specific chemicals were the earliest attempts to develop targeted environmental biosensing. Already in the early 1990s, a Tn21 mercury resistance operon (mer) and naphthalene and salicylate catabolism promoter (nahG) were fused with promoterless luxCDABE to create biosensors for mercury and naphthalene pollution [72,74]. Since those pioneering studies, a plethora of recombinant strains have been created for monitoring toluene and trichloroethylene (TCE), polychlorinated biphenyl (PCB), antibiotics, fast-acting biocide, phenols and heavy metals such as cadmium, mercury, lead, zinc, arsenic copper and cobalt [12,14,16,75,76,77,78].

Interestingly, apart from analyte-specific constructs, several effect-specific lux strains have been developed. These couple the luxCDABE gene to promoters responsible for the bacterial SOS stress response and, thus, aim at the detection of specific cellular toxicity effects or mechanisms underlying the exposure to toxic chemicals, such as the induction of oxidative stress, the synthesis of heat shock proteins, genotoxicity and protein and plasma membrane damage [79,80,81,82].

A comprehensive summary of those constructs and their applications has recently been presented by Woutersen et al. and Eltzov and Marks [12,16].

#### 4.3.2. Fluorescence Methods

In recombinant constructs, the genes encoding fluorescent proteins such as green fluorescence protein (gfp) can be used in place of a lux reporter [13,83]. Several examples have been demonstrated, including biosensing of heavy metals and BTEX [84,85,86,87]. Owning to the recent progress in fluorescent protein technology, the potential major advantage of this strategy is the possibility of creating bacterial strains, each containing several inducible biosensing plasmids. In this scenario, each plasmid would employ fluorescent proteins with different emission spectra [88]. This would allow one to theoretically create multi-analyte or hybrid analyte/effect-specific multi-colour biosensors [13,89]. The implementation of such DNA recombinant technologies on chip-based whole-cell microarray technologies with integrated optic fibres and solid-state optoelectronic elements would open a breadth of multiplexed sensing capabilities [12,13,86].

Unfortunately, the major limitation of fluorescence proteins for online biomonitoring is the considerably slower response time compared to bioluminescent systems [12]. This is because there is a potential lag of several hours between the induction of the promoter and actual synthesis of the protein. The molecules of the latter also need to accumulate in the cell in a sufficient quantity before the fluorescence signal can be reliably detected [12]. Moreover, unlike the lux technology, the significant stability of fluorescent proteins allows their detection even upon their leakage to the medium after cell demise [12]. Since the majority of biosensors are not single-cell analysis technologies and only provide an average signal readout from the entire measurement chamber, this can potentially lead to erroneous results and significant analytical bias.

### 4.4. Practical Aspects of Bacterial Sensing Technologies in Real-Time Water Biomonitoring

Bacteria-based technologies for the online monitoring of toxicity and water quality offer significant operational advantages such as low cost, relative ease of bacteria culture maintenance, rapid analysis time and an ability to utilise genetically engineered strains for analyte-specific detection [12,13,14,15]. Historically anchored successful examples and wide implementation of commercial water biomonitoring systems such as ToxAlarm Toximeter and the recent Microtox^®®^ CTM are a testament to their usefulness in practical deployment scenarios (Figure 2).

However, bacterial-based biosensing systems often need to replace bacteria between sample measurements. The precisely measured number of cells is injected and mixed with water samples in the analysis chamber during each measurement cycle. As a result, the existing commercial biomonitoring systems predominantly use suspension cultures and on-board fermenters. This increases the costs and complexity of the system, as well as generating potentially significant biowaste. Moreover, such systems cannot achieve true flow-through sensing since there is always a delay between the sequence of events that needs to occur to prepare the sample, isolate the analysis chamber and perform the actual sensing. To reduce the costs and complexity of macroscale bacterial fermenters, there have been several attempts to develop online water sensing, employing miniature chip-based bioreactors [14,90,91]. Although this is quite an innovative and promising concept, none of those systems have, thus far, been deployed in the field or tested for long-term operational reliability.

Over the last decade, a tremendous number of prototypes that employ the immobilisation or encapsulation of live microorganisms have been demonstrated [13,14,15,82]. Such techniques can avoid the necessity of maintaining the cultures as well as, in theory, eliminate any possibility of a washing-out bacteria from the analysis chamber, thus affecting the readout and minimising the potential contamination of tested water with transgenic strains. Diverse methods have been proposed in this regard to achieve a solid, semi-solid phase chemical cell immobilisation or entrapment using microfabricated chip-based technologies (biochips) [16,82,90,92,93]. Since immobilised bacteria cells cannot be replenished, unless the entire biosensing element is removed and replaced, most of those technologies have been designed for Point-of-Test, portable and disposable applications for water quality testing [12,13,14,15,16].

There have been, however, several noteworthy and very promising attempts to develop online and flow through biomonitoring technologies using microbial cell immobilisation techniques. In those systems, the immobilisation of bacteria has been predominantly achieved using sol-gel chemistries that embed cells directly on fibre optic elements or form biofilms on different matrixes [94,95,96]. The replacement of sensing elements can be reduced by the utilisation of genetically engineered strains with bioluminescent or fluorescent *switch on*–*switch off* genetic constructs. In such scenarios, the biosensing elements can operate, at least under laboratory conditions, for extended periods and only generate positive signals in the presence of the target analyte. The significant limitation of this design for online deployment is the ability of the bacteria to remain viable and metabolically active when continually exposed to raw, natural water. Interestingly, there is a paucity of comparative data on these important practical aspects that greatly depend on the sensor design, as well as the type of cell immobilising matrix.

**Figure 4:**
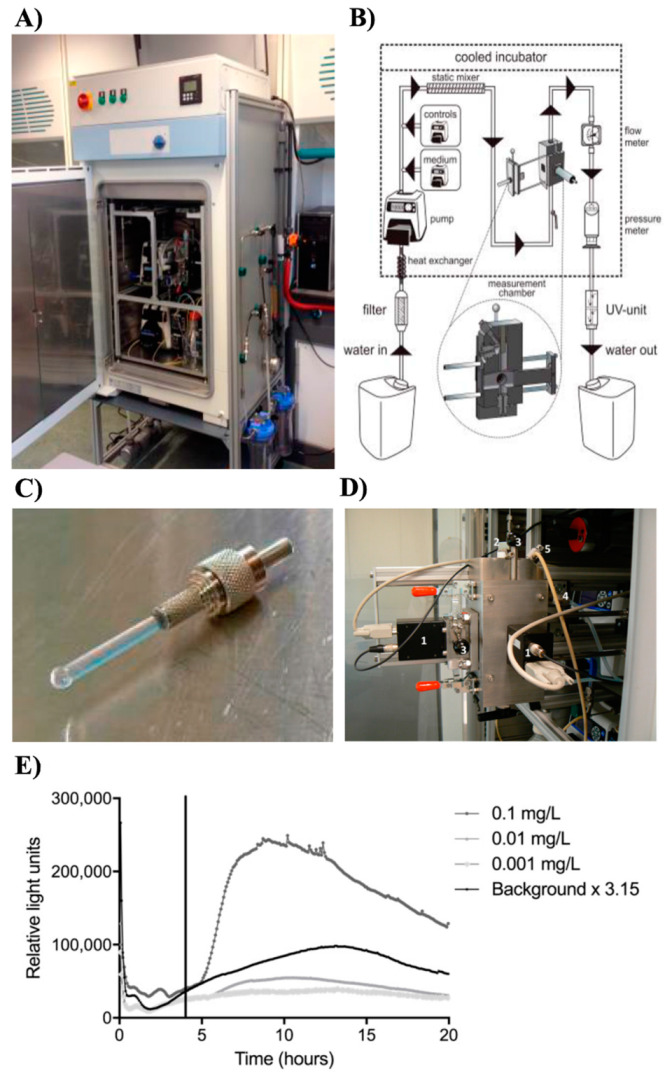
Prototype of bacterial online water quality biosensing system utilising an immobilised recombinant luminescent DPD2794 strain of E. coli that senses occurrence of DNA damage (recA promoter cloned with the luxCDABE genes of Aliivibrio fischeri). (**A**) Photograph of the system deployed for field trials at the water monitoring station in Keizersveer (Hank, The Netherlands) located on the river Meuse, (**B**) A schematic overview of the technical components of the prototype. Note the enlargement of the measurement chamber shown in the inset, (**C**) Photograph of the biosensing element where bacteria are immobilised on the tip of an optical fibre, (**D**) Photograph of the measurement chamber: 1—photomultiplier tube, 2—pH and temperature sensors, 3—light shutters, 4—water outlet, 5—air outlet, (**E**) Validation of background signal and response of the biosensor three concentrations of mitomycin C used as a reference toxicant. Reproduced under the terms and conditions of the Creative Commons Attribution (CC BY) license from [94].

Perhaps one of the best practical validation examples includes a recent prototype of a bacterial online monitor that employed an engineered bioluminescent strain of *Escherichia coli*. It was field tested for potential practical deployment in a water monitoring station Keizersveer (Hank, The Netherlands) located on the river Meuse (Figure 4) [94]. Its performance was also directly compared with the existing installations of animal behaviour-based BEWSs such as DaphTox II (bbe-Moldaenke GmbH, Germany) and the Musselmonitor^®®^ (AquaDect, The Netherlands) that utilise freshwater crustacean *Daphnia magna* and bivalve mussel *Dreissena rostriformis*, respectively [94]. Interestingly, the field trials demonstrated the much lower overall sensitivity of the bacterial online monitoring [94]. This was expected since the system employed only one genetically engineered DPD2794 strain of *E. coli* that sensed the occurrence of DNA damage (recA promoter cloned with the luxCDABE genes of *Aliivibrio fischeri*) [91]. In contrast, animal-based technologies can monitor a wide array of water parameters including toxicants that do not necessarily induce DNA damage. Furthermore, the peak induction of an alert response from the bacterial biosensor was approximately 1–10 h, precluding sensum stricta real-time sensing (Figure 4) [94]. The results of this trial have exposed some of the fundamental limitations of bacterial biomonitors based on genetically engineered strains namely, a very narrow detection window and delayed response time. Moreover, a rarely addressed issue is that genotoxic chemicals often must undergo intracellular metabolic activation before they can induce DNA damage. Therefore, bacterial recombinant biosensors such as the DPD2794 strain without additional modification, including, for instance, the addition of eukaryotic cytochrome P450 genes, are inherently incapable of accurately detecting the genotoxic effects of most chemicals [16].

Further improvements could potentially be achieved with multiple recombinant microorganisms capable of detecting a broad variety of chemicals [97]. Recently, live-cell microarray technology that utilises twenty recombinant bioluminescent bacteria to detect and classify a range of toxicants has been demonstrated [98]. There is hope that the latter technologies, in conjunction with the biopatterning, emerging microfluidic Lab-on-a-Chip technologies and miniaturised optoelectronics, could be prospectively embedded into the integrated online synoptic systems [92,99,100].

Yet another non-trivial problem highlighted by the above field trial was the significant fouling of the sensing element that severely impaired the measured intensity of the luminescent signal. This required manual daily cleaning of the sensing element, thus making it laborious and impractical for long-term online deployments [94]. In this regard, applications of non-optical technologies, such as MFCs, could perhaps be a more advantageous approach in continuous monitoring scenarios [41,67,68]. Although MFCs can reportedly provide the long-term stability of the biosensing elements, there is a paucity of data on their performance in actual comparative field trails, such as the one demonstrated by Woutersen et al. [94]. Accordingly, it is unknown if bio- or chemical fouling of the anode exposed to natural water will be problematic as well as if the bacterial biofilms in the MFCs will remain viable and metabolically active when continually exposed to raw water.

In conclusion, despite a few very innovative online monitoring prototypes demonstrated and validated under nominal laboratory conditions for the detection of pesticides, heavy metals and PCBs and other pollutants, none of them have thus far been commercialised or deployed in routine applications. This demonstrates that despite two decades of active research and development worldwide, there are non-trivial and practical operational problems remaining that need to be resolved for these systems to mature. At present, the existing deployments still utilise conventional biomonitoring systems, such as ToxAlarm Toximeter and the Microtox^®®^ CTM, and despite lacking more recent biosensing innovations, these remain examples of rugged and reliable online biomonitors. It is our hope that the development of next generation systems will be primarily driven by practical deployment considerations and based on more stringent, comparative evaluations under extended field trials.

## 5. Cyanobacteria Biosensing Technologies

Cyanobacteria, often called blue-green micro-algae, are prokaryotic microorganisms that exhibit photosynthetic activity thanks to membrane-bound chlorophyll [21,101]. They were historically one of first prokaryotic organisms used in attempts to develop whole cell biosensing of toxicants in water. Their major applications have been initially aimed at the detection of photosynthesis inhibitors, such as herbicides [21,101,102]. Cyanobacteria species *Synechocystis* sp. and *Synechococcus* sp. were demonstrated in several biosensing applications using respirometry analysis to measure the effects on aerobic and/or anaerobic metabolism, nitrogen fixation as well as the fluorometric and amperometry detection of photosynthesis inhibition [21,101,102,103,104]. Subsequently, transgenic technologies were allowed to create multiple stable strains, each detecting specific metal ions such as cadmium, zinc, copper, mercury, cobalt, chromate, thallium and nickel [103]. This was achieved by fusing metal-responsive promoters with genes encoding either firefly luciferase (luc), bacterial luciferase (luxAB) as well as genes of green-fluorescent protein (gfp) [21]. Bioluminescent strains were developed for biosensing the presence of nitrates and heavy metals in water [105,106]. This was achieved by using a tac promoter cloned with bacterial luciferase [107]. The luminescence and fluorescence cyanobacterial biosensors can provide a simple, rapid and economical detection of specific toxicants [104]. However, they do require transgenic organisms that are tightly regulated due to the potential of their release to the environment to be utilised. Moreover, since transgenic biosensing elements are inherently analyte-specific, multiple strains would have to be utilised in any practical deployment of a water biomonitoring system to simultaneously provide detection of diverse toxicants. The presence of other chemicals that fall outside an analyte-specific strain would not be detected. This adds significant complexity since the detection of multiple independent bioluminescence strains needs to be monitored in real time as well as combined with fluorimetry or amperometric measurements of general photosynthetic activity to provide a global toxicity readout. Similarly, to bacteria-based biosensors, significant technical complexity is required to enable the immobilisation, encapsulation or reliable containment of cyanobacteria cells for continuous analysis [104,106,108]. As of today, no practical deployment of water biomonitoring systems has been reported with cyanobacteria-based biosensing technologies [21].

## 6. Algal Biosensing Technologies

Green algae are photo-autotrophic eukaryotic organisms characterised by the presence of a double-membranous nucleus and organellar intracellular compartmentalisation. They can be broadly classified into (i) macro-algae, a term used for seaweeds and other benthic macrophytes that possess highly specialised tissue structures; and (ii) micro-algae, used as a collective term to describe unicellular, microscopic photosynthetic eukaryotes that form freshwater and marine phytoplankton [21,109]. Many marine and freshwater species of micro-algae have been explored in ecotoxicity testing and potential water biosensing and biomonitoring technologies. In this regard, the *Chlorella vulgaris* and *Selenastrum capricornutum* remain the most common unicellular photosynthetic organisms employed [26,109,110]. Whole-cell algal systems can potentially utilise diverse endpoints for the real-time detection of chemicals including alterations in cell viability, cell proliferation, the inhibition of selected enzymatic activity or the inhibition of general metabolic indices such as respiration and photosynthesis. To this end, diverse technologies have been employed in a plethora of published prototypes, but, in general, the most practical from the practical deployment are the time-tested solutions that include the following:

### 6.1. Respirometry

A technology utilising dissolved oxygen sensors, built into an enclosed and fixed volume measuring chamber, which is filled with a water sample and a precisely calculated number of algal cells. Such systems can measure both aerobic metabolism (decrease in oxygen content without illumination) as well as photosynthetic activity (increase in oxygen content when illuminated with actinic or blue spectrum of light source). The continuous monitoring of dissolved oxygen levels can be performed using optical fluorescence quenching or, more commonly, amperometric Clark electrode-based sensors [111]. Several automated systems utilising fibreoptics to deliver an actinic light source and small flow-cell reaction chambers have been prototyped to measure the impacts of toxicants on photosynthetic activity in real-time [111,112]. Such systems were, however, not designed as online water biomonitoring technologies and predominantly aimed at laboratory ecotoxicity tests with an average measurement time per sample of approximately 10 min (Figure 1). More recently, several innovative microfabricated devices were presented where the oxygen generated by immobilised cells of micro-alga *Chlorella vulgaris* was monitored amperometrically using transparent ITO (indium tin oxide) electrodes and motility biosensing [113]. Such miniaturised and disposable biosensors were demonstrated in rapid water toxicity testing, but thus far, have not found any practical applications in online water biomonitoring systems.

### 6.2. Fluorimetry

This technique measures very weak fluorescence emissions of chlorophyl a when illuminated in situ with a broad spectrum or blue wavelength (470 nm) of light. The chlorophyl emissions collected at wavelengths of 690 and 740 nm are characteristic for activity of Photosystem II (PSII) and Photosystem I (PSI), respectively [114]. Fluorometric measurements have indeed gained significant applications in an assessment of biotic and abiotic plant stressors [115]. Analysis of the photosynthetic activity using chlorophyl fluorescence kinetics is a non-invasive, label-free and rapid method used to indicate the changes in functionality of chloroplast photosystems. It can also be well correlated with the algal cytotoxic effects induced by pollutants. Recently, a pulse amplitude modulated fluorometry (PAMF) was introduced [116,117,118,119]. Apart from standard measurements, this technology includes measurements of chlorophyl fluorescence upon saturating light pulses [118]. It is today widely regarded as the gold standard fluorometric method to precisely and reliably assess PSII quantum efficiency [118,119]. Namely, inhibition of the photosynthetic activity directly correlates with a decrease in PSII quantum yield, and this endpoint is particularly sensitive for the detection of herbicides that predominantly affect the functionality of the PSII. Reportedly, in some algal species, PSII quantum yield can also be used to sense acetylcholinesterase inhibiting insecticides and some heavy metals [114,115,118,119]. Interestingly, there are significant differences in inter-species sensitivity between micro-algae exposed to the same type and even concentration of pollutants when measured using PAMF [109]. This adds to the complexity of using PAMF in online water biomonitoring systems, since the selection of bioindicator species is not universally standardised.

The majority of historic algae-based biosensor prototypes have exploited chlorophyll fluorescence as the measurable endpoint performed in flow cells under continuous perfusion. Such systems are usually based on macroscale perfusion chamber-based designs, the encapsulation and immobilisation of algae in diverse gels or silica membranes and, more recently, portable and miniaturised microfluidic-chip-based technologies [104,120,121,122,123]. The latter emerging technologies were also used to develop integrated multiparameter measurements for the in situ portable biosensors including electrochemical, algae fluorescence, dissolved oxygen O_2_ and pH endpoints [124].

### 6.3. Chlorophyl Fluorescence Imaging

The *in situ* imaging of chlorophyl fluorescence at 680 nm upon excitation with a blue spectrum (488 nm) of light is a relatively new, non-invasive and rapid technique to assess phytotoxicity [125,126,127,128]. The imaging paradigm enables the acquisition of time-resolved images of spatio-temporal variations in photosynthetic activity using cooled digital cameras [126]. Accordingly, it has been increasingly applied as an endpoint in plant ecotoxicology and plant biology [125,126,127,128,129]. Recently, several studies have demonstrated this technique for high-throughput algae cytotoxicity tests on 96-well multi-plates using automated IMAGING-PAM M-Series fluorimetry system (Heinz Walz GmbH, Germany) [126]. The advantages of this approach for chemical water analysis include the lack of any requirement for stains or extensive sample processing, rapid data acquisition and ultra-high throughput capabilities [109,118,119,126,127]. Although the pulse amplitude modulated fluorimetry is a superior technique to allow for PSII quantum efficiency to be calculated, rapid chlorophyl fluorescence imaging can be also performed using conventional fluorescent microscopy, two-photon excited fluorescence lifetime imaging (TPE-FLIM) and laser-scanning or imaging cytometry systems. To the best of our knowledge, this technique was demonstrated only in accelerated ecotoxicity testing and has not been tried in any prototypes of online water biomonitoring systems [109,118,119,126]. This is a very interesting notion for further exploration. Considering its ultra-high throughput, one could envisage, for instance, a combination of a robotic liquid handling or microfluidic LOC technologies to mix water samples with a precisely measured number of algal cells. The subsequent PAM fluorescence imaging could achieve near real-time biosensing capabilities at a high-throughout. In a sense, such a system could be built based on existing know-how and the technologies used routinely in drug discovery applications that exploit robotic liquid handing and automated high-content imaging.

### 6.4. Practical Aspects of Algal Technologies in Real-Time Water Biomonitoring

Despite the significant potential of algal biosensing methods for the water quality assessment outlined above, one of their significant limiting aspects is a necessity to utilise the entrapment and/or immobilisation of cells to maintain a precise and unchanging number of cells between measurements and to prevent their diffusion and leaching during analysis. For algal cells in suspension, this has usually been achieved in optical flow-through chambers or dispensed into conventional multi-well plates [26]. Diverse methods have also been proposed to achieve solid phase immobilisation using gel entrapment, microencapsulation and a forced formation of biofilms in different matrices such as poly(vinyl alcohol) (PVA), polysulfone (PSU), porous silica (e.g., quartz microfibre filters) and cellulose derivatives [21,104,111,122]. Algal cells must be, however, replaced after each measurement and with solid-phase or gel-based matrix substrata; this is impossible to achieve because they irreversibly bind cells, thus preventing their rapid replacement. Although such technologies can be suitable for handheld portable and disposable field biosensors, they are not optimal solutions for online water biomonitoring paradigms [21,104].

As a result, the majority of recent studies utilised microfluidic Lab-on-a-Chip (LOC) technologies. They offer the ability to perform continuous microperfusion bioanalysis under low Reynolds numbers and to implement diverse microfabricated functionalities such as mechanical dams, sieves for cell entrapment and micro-scale optical chambers [124,130]. Moreover, LOC facilitates the integration of many active and miniaturised analytical components such as micro-valves, solid state light emitting diodes and electrodes. A highly innovative avenue with an LOC system that has not been thus far explored is the utilisation of microflow cytometry (µFC) [131,132,133]. This technology represents a vastly miniaturised version of conventional FC. Its sensitivity falls within the specifications of conventional cytometric systems, but this next-generation technology promises greatly reduced equipment costs and portability. Most importantly, as only ultra-low cell numbers and operational reagent volumes are required in microflow cytometry, functional cell studies in real-time appear to be possible [133]. As mentioned before, FC has been successfully demonstrated in rapid algal cytotoxicity assays using fluorescent viability probes [33,134]. By virtue of cell counting abilities and its common 488-nanometer excitation wavelength, it is also applicable to perform in-flow measurements of chlorophyl fluorescence at the single-cell level. Multiparameter flow cytometric analysis of the inhibition of the chlorophyl fluorescence can also be combined with measuring global esterase activity as a marker of cell health using a cell permeable fluorescein diacetate (FDA) probe and plasma membrane permeability as markers of cell death using markers such as DRAQ7 or SYTOX stains [33,134]. The automation and real-time sensing of flow cytometric parameters could be achieved with µFC, including automated sampling, staining and time-resolved cytometric analysis.

To this day, many prototypes of miniaturised algal biosensors have been demonstrated, some with very innovative designs and promising analytical characteristics. Many nascent technologies were also successfully validated in ecotoxicity studies aimed at the detection of herbicides, insecticides, heavy metals and industrial solvents. However, despite their potential for portable field use and even online water biomonitoring, the Algae Toximeter (bbe Moldaenke) remains the sole example of a successful and practical deployment of algal biosensing technology for near real-time sensing of water quality [26].

## 7. Biosensing with Vertebrate Cells

Automated technologies that utilise artificially grown layers of adherent vertebrate cell lines as biosensing elements have been developed for a wide variety of high-throughput applications in drug discovery, predictive human toxicology and, more recently, ecotoxicity testing [135,136,137]. Quantitatively measured outputs usually include (i) respirometry parameters, such as acidification (an indicator of anaerobic respiration) and oxygen consumption (an indicator of aerobic respiration) and, (ii) more recently, impedance measurements (Figure 1). The later technique, pioneered by Giaever and Keese, is commonly referred to as the electrical-cell-impedance-system (ECIS) or impedance spectroscopy [135,136,138]. It has been extensively demonstrated and validated in high-throughput cell cytotoxicity studies in both human and environmental toxicology [137,139,140]. The ECIS principle involves growing a monolayer of adherent cells on microelectrode arrays. The latter detects local changes in the ionic environment at the electrode-medium interface. Ionic changes are affected by the presence of adherent cells and the resulting change in the impedance value read from the electrodes [141,142]. Cytotoxic effects as well as any changes in cell proliferation and, thus, cell numbers alter the structure of the growing monolayer interacting with electrode arrays. This is, in turn, reflected by the detectable changes in electrical impedance. The ECIS technology allows a label-free, non-destructive and real-time analysis of cell viability and proliferation [141,142,143]. It provides several analytical advantages such as (i) a lack of any fluorescent labels and imaging, (ii) the ability to perform real-time kinetic monitoring of cells, (ii) ease of operation and (iv) straightforward automation [140,141]. Commercial examples of existing ECIS systems that are widely used for laboratory toxicity tests include the xCELLigence (Agilent, Santa Clara, CA, USA), Maestro platform utilising CytoView-Z impedance plates (Axion BioSystems, Atlanta, GA, USA) and Bionas 2500 (Bionas, Rostock, Germany) [142].

The utilisation of vertebrate cell biosensors including ECIS technology for the monitoring of water quality has, thus far, been a niche application with only a very small number of prototypes presented as compared to bacterial and algal sensors [139,140,143,144]. The theoretical advantages of mammalian cell biosensors include a real-time response to a wide variety of toxicants in real-time. This is advantageous when compared to cell-free biosensing methods such as immunosensors, enzymatic and DNA biosensors. When utilising human cell lines, one can also postulate that their responses might also be better applicable to modelling the potential impact of toxicants on human health [143].

However, the exploitation of mammalian cell lines, including popular bovine pulmonary artery endothelial cells (BPAECs) or bovine lung microvessel endothelial cells (BLMVECs) for online and continuous monitoring of water quality, presents with very significant obstacles. First and foremost, the culture of any mammalian lines in pure water is impossible since, to support their survival and growth, they require specialised culture media as well as a 5% carbon dioxide environment at 37 °C. This essentially precludes any online water biomonitoring applications without elaborate sample pre-processing [139,145,146]. For example, to enable water to be tested, it must be mixed with concentrated or powdered cell culture media to re-constitute it with the correct osmolarity, pH and balanced nutrients [139]. Although such pre-processing steps can be, in theory, automated, the added complexity might be cumbersome for practical deployment in biomonitoring systems. Moreover, the added chemical complexity of cell media may significantly affect the results due to interactions between the toxicants and constituents of the media. Interestingly, one study has also demonstrated that diluted wastewater (1:10–1:10,000) could be used with L6 rat myoblasts cells for real-time respirometry and impedance analysis [144].

The problems associated with mammalian cells fuelled the development of fish gill cell lines as well as a primary FIsh Gill Cell culture system (FIGCS) for ecotoxicity testing and their potential applications in water biomonitoring studies [143,147,148]. The rainbow trout gill epithelial (RTgill-W1) cell line has gained significant popularity. It is an immortalised fish cell line [148]. Those cells are characterised by their slow proliferation capacity and can be reliably grown in monolayers at ambient carbon dioxide levels from 6–20 °C for up to 78 weeks without any media changes [143,145,148]. They are also reportedly sensitive to a broad spectrum of toxicants at concentrations that impact human health [149]. Those characteristics are suitable for ECIS systems, opening avenues for inexpensive, low maintenance cell-based biosensors [143]. Despite the above advantages, however, the RTgill-W1 cells still require a specific L-15 culture medium that must be reconstituted from a powdered mix with water samples to be tested [143,145].

The above limitations of RTgill-W1 cells as a biosensing element led to the development of the primary FIsh Gill Cell culture system (FIGCS) [147,150,151]. FIGCS cells can be cultured at ambient temperatures with no carbon dioxide supplementation. The fact that they reportedly tolerate the application of raw river water on the apical surface, thus mimicking the physiological functionality of live fish, is particularly critical for water biomonitoring paradigms [150,151]. When exposed to uncontaminated natural waters, the FIGCS showed no cell viability loss following a 24-h incubation. Moreover, the FIGCS has been successfully transported for field testing (approximately 1000 km during a 30-h period) during both laboratory and field-based heavy metal contamination monitoring of rivers [150,151,152]. The development of innovative cell-based systems, such as the FIGCS, offers tremendous opportunities for whole-cell-based biosensors that can exploit ECIS, respirometry as well as fluoresce viability probes as endpoint readouts.

## 8. Limitations of Live-Cell Online Biomonitoring in Practical Deployment Scenarios

Despite the potential advantages of whole-cell systems in laboratory and portable Point-of-Test applications, they have numerous disadvantages for online water quality biomonitoring. Apart from the technology specific examples provided in the preceding sections, we will now briefly highlight the most critical aspects that need to be considered when designing systems for practical deployment. Surprisingly, those non-trivial aspects are rather infrequently discussed in the contemporary engineering literature on live-cell water biosensors.

### 8.1. Non-Quantitative Nature

With limited exceptions, such as, for instance, microarrays of immobilised transgenic bioluminescent bacterial strains, each detecting the presence of a specific toxicant, the very nature of cell-based biosensing is non-specific with regard to the type of contamination and, in the majority of cases, also concentrations of the chemicals [25]. In other words, this type of technology does not provide any specific information about the actual chemical composition of the water samples. This is perhaps one of the biggest criticisms of all the BEWSs, including the ones using analysis of animal behaviour [1,2,20]. However, their often-overlooked advantages are a synoptic early warning against sudden and global changes in parameters that can be considered harmful to live organisms and, in translation, also to human health [1,2,20]. Cell-based biomonitoring technologies can, thus, act as supplementary on-line applications to the existing chemical analysis regimens. Their successful deployments were demonstrated by established examples that are proven to be very reliable in many worldwide installations [25].

### 8.2. Analysis Time

As discussed in the preceding sections, many prototypes of biosensing technologies do not offer a true real-time sensing capability, defined as the continuous sensing of water parameters [25]. At present, only the Microtox^®®^ CTM system is capable of real-time biosensing with measurements performed on average every ten seconds. Most cell-based technologies developed thus far offer near real-time analysis with samples processed, at best, every 15–30 min. Furthermore, many bacterial biomonitoring prototypes utilising genetically engineered strains demonstrate peak response times between 1 and 10 h [94]. This is generally in stark contrast to commercial examples of BEWSs based on animal behaviour, where sensing always occurs in real-time. However, it needs to be remembered that the near real-time capacity is not necessarily a real disadvantage for practical deployment [25]. Considering that the dead volume in pipes of the drinking water purification plant is very large, the total replacement of water in the system in any case will not likely not occur in seconds. Systems that feature a delayed response time lasting many hours cannot be considered as biomonitors for practical deployments.

### 8.3. Maintenance of Cell Cultures

The solid phase bacterial and algal biosensors have very limited applicability in online water biomonitoring systems. As a result, all the practical deployment examples of commercial cell-based biomonitoring technologies, such as Microtox^®®^ CTM, ToxAlarm Toximeter and Algae Toximeter II, are equipped with on board fermenters to provide a continuous culture of cells for biotests. This is certainly one of the weak links of all cell-based technologies, since any contamination of stock culture or technical failure in the automated culture apparatus will immediately put a hold on their operation [25]. The redundancy engineering can include dual, independent fermenters, albeit at an increased cost and complexity of such systems. The issues related to the longevity of the cell cultures and, hence, the service time required for replenishing cells, reagents and media, are also non-trivial.

### 8.4. Sterilisation Protocols

Cell-based systems require sterile environments to maintain the stock cultures. The sterilisation of the main fermenter tanks and associated components is periodically needed and can be, to a large extent, automated, using contemporary advances in miniaturised laboratory automation and mechatronics. However, historical deployment experiences with previous generations of bacterial online biomonitoring stations on the river Rhine demonstrated that the contamination with native bacterial species and the significant growth of natural water bacteria in the tubing and detection chambers was indeed a cumbersome problem [11,25]. The postulated solutions included implementations of stringent, daily sanitation protocols and the automated pre-filtration of water samples [11,25].

### 8.5. Pre-Processing of Water Samples

If implemented due to the significant load of native microorganisms and/or excessive abiotic particulate matter present in the tested waters, the filtration cannot change the chemical composition of the water [11,25,94]. Complicating the complexity of this topic is the fact that anthropogenic pollutants adsorb to suspended nano- and micro-particulate matter and this is indeed often their major route of exposure. Hence, the water filtration can introduce a significant analytical bias and effectively create false negative readouts [25]. At present, those aspects have not been satisfactorily resolved for conventional and miniaturised biosensing technologies.

### 8.6. Waste Disposal

In many of the technologies developed thus far, fresh batches of cells are required for each independent measurement. This creates a potentially significant load of biological waste accumulating over time when sensing occurs continuously. With systems utilising genetically engineered microorganisms or species that are not native to the geographical place of installation, very stringent biosecurity measures must be employed to avoid the contamination of the ecosystem. In this regard, automated waste collection, secure storage and certified decontamination protocols must be built in. They must be also continually assessed for meeting compliance requirements, since any containment breaches can have very wide and negative ramifications.

### 8.7. Thresholds of Sensitivity

The sensitivity of biosensors and their speed of response to a spectrum of chemicals at toxic concentrations is critical for any successful online biomonitoring deployment [2,25]. Monitoring systems to be deployed at catchments of drinking water, drinking water treatment as well as aquaculture plants must have a wide spectrum of sensitivity to diverse substances and be carefully validated in laboratory conditions. However, the sensitivity of biosensors varies significantly with cell types/species. The latter have been mostly selected empirically and tested with limited numbers of chemicals across nominal concentration ranges. For instance, algae biosensors are inherently more sensitive toward the toxicants inhibiting photosystem activity, such as herbicides. At the same time, major intra-species variability in algal sensitivity to the same chemicals has been widely reported. This can create a situation where two biomonitoring systems equipped with a different species of algae as biosensing elements will provide vastly different readouts. In general, there is a notable paucity in large comparative trials to evaluate different biosensing technologies and estimate sensitivity thresholds for the most common practical deployment scenarios [2,25]. It is imperative that the validation of biosensing prototypes and new test installations considers the appropriate local regulations and water quality directives published by national water management bodies [2,25].

### 8.8. Reliability of Alarm Events

In all the online monitoring applications, alarms are automatically triggered when the computer-controlled biosensing detects statistically significant alterations in the pre-set thresholds [2]. This event potentially indicates a presence of chemical pollutants. The alarm events are related to the thresholds of sensitivity discussed in the preceding section, but the evaluation of their reliability is usually a long-term and deployment-specific process that includes estimating numbers of false positive and negative alarm events [2]. Alarm events can stem from diverse and unexpected changes in water parameters such as dissolved oxygen, dissolved carbon, pH, particulate matter load and a potential contamination or infection of fermenters and fluidic components with pathogens. Some of these can be continuously monitored with built-in chemical sensors but others may be very difficult to detect and validate, thus impacting the installation reliability [2]. In this regard, some cell-based biosensors, due to the above-described operational complexity, may be more prone to reliability issues compared to animal behaviour-based BEWSs.

## 9. Conclusions and Future Outlook

Live-cell-based biosensing of water quality parameters is a viable method that can supplement conventional chemical analysis test strategies. Although biological methods do not provide analyte-specific data about the actual composition or concentration of toxicants, they can be successfully implemented in early warning systems against sudden contamination. Compared to paradigms based purely on chemical analysis, biological sensing with live cells and whole intact aquatic animals also provides important data on the biologically specific impact of chemical pollution. When applied to continuous, real-time analysis, biomonitoring can also fill the gaps associated with a very limited sampling frequency of chemical analysis. Recent years have brought a significant number of innovations in whole cell biosensors, such as tremendously diverse cell immobilisation techniques, the implementation of microfluidic chip-based technologies, miniaturised optics and electronic transducers, real-time embedded computing as well as genetically engineered cell systems with bioluminescent and fluorescent reporters.

However, despite the potential of predominantly algal and bacterial biosensing methods for portable Point-of-Test devices, significant obstacles remain to be overcome for the next generation of online monitoring applications. They include technical aspects such as the long-term resistance to biofouling and the corrosion of sensing elements, increasing the single-to-noise ratio from miniaturised transduction elements as well as the automation of a long-term culture and the immobilisation of cells. The latter methods must prevent the leakage of genetically engineered bioreporters into the environment but at the same time allow cells to be rapidly replaced after each analysis cycle.

Apart from these technical aspects, the cell biosensing elements, with a few notable exceptions, are generally not analyte specific. Moreover, as discussed for algal biosensors, a significant inter-species heterogeneity of responses to chemicals exists. Those issues are not trivial, but future innovations and technology developments will likely address them and focus on multispecies systems and perhaps even hybrid technologies utilising a plethora of promising advances in cell-free water biosensing technologies such as miniaturised surface acoustic waves (SAWs), surface plasmon resonance (SPR), surface enhanced Raman spectroscopy devices, as well as acoustic immunosensors and enzymatic and DNA biosensors [2,8]. Hybrid technologies will surely increase the complexity of the biomonitoring systems but, at the same, can provide a more robust multi-analyte sensing capability. Some of the existing obstacles will likely be overcome using technical advances in miniaturisation such as respite growing micro-electro-mechanical systems (MEMS) and even nanotechnology. We also postulate that practical implementations of cell-based biosensors should be coupled with existing animal behaviour-based biomonitoring technologies to dramatically improve not only the sensitivity and specificity but also the resilience of the future online devices deployed in the synoptic monitoring of water quality [2].

Lastly, as postulated recently for animal behaviour-based biomonitors, there is a need for standardised strategies and common approaches to validate sensitivity in large multi-centre trials. Despite a tremendous number of existing prototypes and small-scale laboratory studies, there is a paucity of data on practical validations to demonstrate if their sensitivity and reliability adhere to the existing water quality testing guidelines [2]. The lack of such studies makes comparative analysis, cross-validation of different new designs and making recommendations very difficult. Efforts should, therefore, be increased to harmonise the validation strategies for water biosensing coupled with the development of guidelines on deployments as part of wider country-specific water monitoring test strategies.

In conclusion, despite existing limitations, the practical potential of cell-based biosensors in the synoptic, online screening of water quality is well anchored in the successful deployment examples of conventional systems. The next generation of miniaturised biosensing cell-based technologies is budding. Despite the further advances and developments required, they can indeed form a valuable part of new and futuristic bioanalytical strategies deployed for real-time sensing of water quality.

## Data Availability

Not applicable.
